# Effect of silver nanoparticles on *Candida albicans* biofilms: an ultrastructural study

**DOI:** 10.1186/s12951-015-0147-8

**Published:** 2015-12-15

**Authors:** Humberto H. Lara, Dulce G. Romero-Urbina, Christopher Pierce, Jose L. Lopez-Ribot, M. Josefina Arellano-Jiménez, Miguel Jose-Yacaman

**Affiliations:** Department of Physics and Astronomy, The University of Texas at San Antonio, One UTSA Circle, San Antonio, TX 78249 USA; Department of Biology and South Texas Center for Emerging Infectious Diseases, The University of Texas at San Antonio, San Antonio, TX 78249 USA

**Keywords:** Electron microscopy, *Candida albicans*, Silver nanoparticles, Filamentation, Biofilm formation, Cell-wall

## Abstract

**Background:**

*Candida albicans* is the most common pathogenic fungus isolated in bloodstream infections in hospitalized patients, and candidiasis represents the fourth most common infection in United States hospitals, mostly due to the increasing numbers of immune- and medically-compromised patients. *C. albicans* has the ability to form biofilms and morphogenetic conversions between yeast and hyphal morphologies contribute to biofilm development and represent an essential virulence factor. Moreover, these attached communities of cells are surrounded by a protective exopolymeric matrix that effectively shelters *Candida* against the action of antifungals. Because of dismal outcomes, novel antifungal strategies, and in particular those targeting biofilms are urgently required. As fungi are eukaryotic, research and development of new antifungal agents has been difficult due to the limited number of selective targets, also leading to toxicity.

**Results:**

By microwave-assisted techniques we obtained pure 1 nm spherical silver nanoparticles ideal for their potential biological applications without adding contaminants. A phenotypic assay of *C. albicans* demonstrated a potent dose-dependent inhibitory effect of silver nanoparticles on biofilm formation, with an IC_50_ of 0.089 ppm. Also silver nanoparticles demonstrated efficacy when tested against pre-formed *C. albicans* biofilms resulting in an IC_50_ of 0.48 ppm. The cytotoxicity assay resulted in a CC_50_ of 7.03 ppm. The ultrastructural differences visualized under SEM with silver nanoparticles treatment were changes in the surface appearance of the yeast from smooth to rough thus indicating outer cell wall damage. On the fungal pre-formed biofilm true hyphae was mostly absent, as filamentation was inhibited. TEM measurement of the cell-wall width of *C. albicans* after treatment resulted in significant enlargement (206  ±  11 nm) demonstrating membrane permeabilization.

**Conclusions:**

Our results demonstrate that silver nanoparticles are potent inhibitors of *C. albicans* biofilm formation. SEM observations are consistent with an overall loss of structure of biofilms mostly due to disruption of the outer cell membrane/wall and inhibition of filamentation.TEM indicates the permeabilization of the cell wall and subsequent disruption of the structural layers of the outer fungal cell wall. The anti-biofilm effects are via cell wall disruption.

**Electronic supplementary material:**

The online version of this article (doi:10.1186/s12951-015-0147-8) contains supplementary material, which is available to authorized users.

## Background

*Candida albicans* (*C. albicans*) is a pleomorphic fungus which is a normal commensal of the gastrointestinal microbiota in healthy individuals; however, as an opportunistic pathogen, *C. albicans* is the most common etiological agent of candidiasis, now the fourth most frequent infection in the US hospitals [1], mostly due to the increasing numbers of susceptible compromised patients that are at risk of this systemic fungal infection [2–4]. One important factor that contributes to the pathogenesis of candidiasis is biofilm formation, as *C.albicans* has the ability to form biofilms on both inert and biological surfaces. In particular, indwelling prosthetic devices and catheters possess an optimal surface for *Candida’s* biofilm formation [5] and *C. albicans* is one of the most common pathogens isolated in catheter-related blood stream infections resulting in high morbidity and mortality rates [2]. These biofilms are typically surrounded by an exopolymeric substance (EPS) matrix [6] which effectively shelters the fungal cells against adverse environmental conditions, including host defense mechanisms and the action of antifungals [7]. Moreover, *Candida* biofilm formation is clinically important as sessile communities of yeasts are the reservoirs originating the spread of fungal infections [8]. It has been demonstrated that morphology (yeast to hypha morphogenetic conversions) plays a very important role in biofilm formation and also represents a critical virulence factor [9]. The functions of filamentous hyphae are adhesion, invasion to biotic or abiotic surface, reinforcement of the fungal colony, nourishment and the construction of a tridimensional designed formation [10].

Research and development of new antifungal agents is complicated by the paucity of selective targets, since fungi are eukaryotic cells. This fact is also responsible for high levels of toxicity displayed by some of the current antifungals, particularly the polyenes and to a lesser extent the azoles [11]. In addition, drug resistance of *C. albicans* against antifungals such as azoles and echinocandins represents an increasing problem. Formation of biofilms further complicates treatment, as sessile cells within these biofilms have the ability to resist drug concentrations even 1000-fold higher than the IC_50_ reported for the planktonic yeasts [6, 12]. Together, all these factors combined are responsible for clinical failures and high mortality in patients with invasive candidiasis [13]. Therefore, novel antifungal and antibiofilm drugs against these unmanageable infections are urgently needed [7, 11].

With the advances on Nanotechnology, silver nanoparticles (AgNPs) have demonstrated to be broad-spectrum bactericidal [14], virucidal [15–17] and fungicidal [18–20]. In recent studies, AgNPs have also proven its efficacy against *C.albicans* [21–24] by disrupting the membrane potential and forming pores causing ion leakage and other materials [20], inducing apoptosis [19] and causing ultrastructural changes [18]. In the present study we describe the potent activity displayed by AgNPs against *C. albicans* biofilms and report on the resulting ultrastructural changes, both at the cellular level and on the overall biofilm structure. In this article we describe the synthesis of pure positively charged AgNPs without added chemicals, and more importantly, the AgNPs effects on inhibition of filamentation and biofilm formation of *C. albicans.*

## Results and discussion

### Generation and characterization of AgNPs

**Fig. 1:**
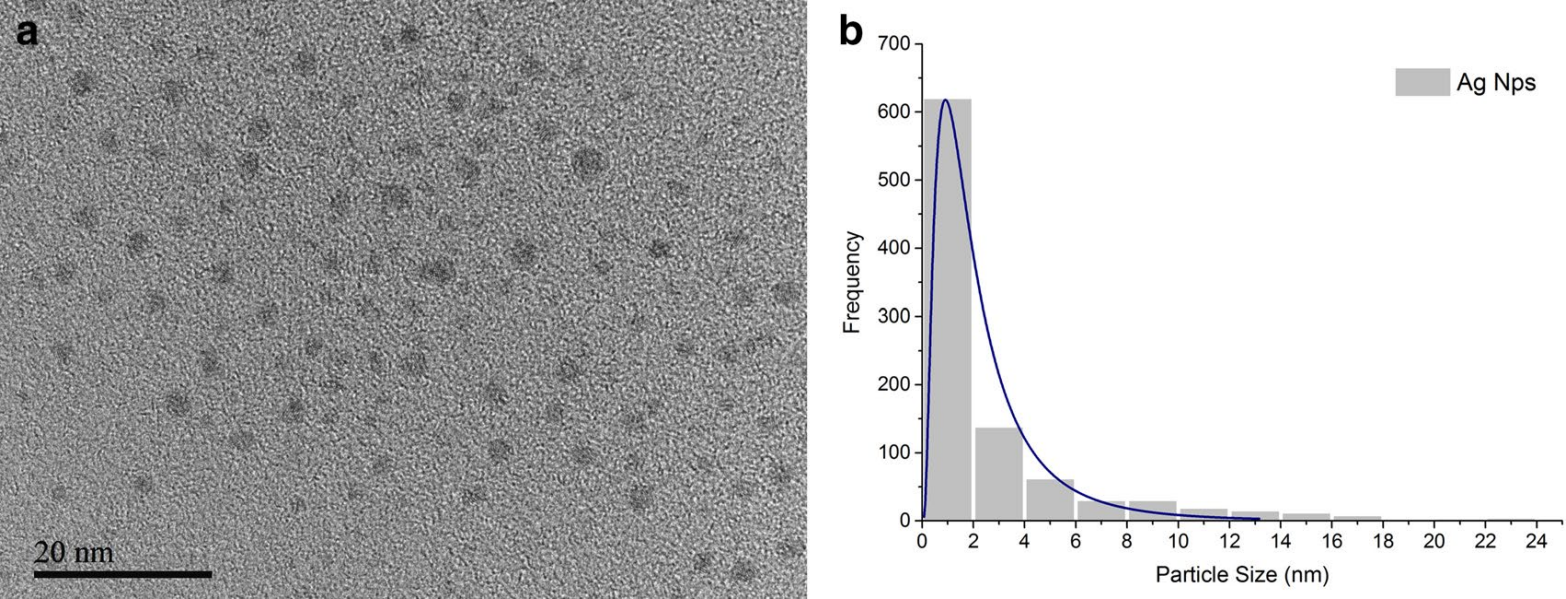
**a** TEM micrograph of AgNPs showed spherical shape nanoparticles. **b** Log-normal size distribution histogram shows the average AgNP size as being approximately 1 nm

AgNPs were prepared in the laboratory by microwave-assisted techniques that have the advantage to achieve fast heating and reaction completion to produce large-scale metal nanoparticles. This technique is a good alternate methodology to synthesize metallic nanoparticles obtaining an efficient control of nanoparticles size distribution (Additional file 1), and more importantly for biological purposes as it does not involve the handling of potentially contaminants or cytotoxic reducing agents [25]. Throughout the synergy of microwave-reactor methodology applied to AgNO_3_ in the reaction process, reduction takes place producing reducing species eliminating the requirement of additional reductors, consequently excluding further contamination sources [26]. With this methodology the reaction can be regulated throughout the control of temperature, applied power and reaction time, thereby leading to high-yielding, clean, scalable reactions and precise size distributions of AgNPs without contaminant on biological tests [26]. The Zeta potential value increased with time from −2.9 to +13.4 mV over a 120 h time period. This shift to positive Zeta potential demonstrates the adsorption of cations onto particles from the solution tested [27]. This Zeta potential result suggests that our AgNPs become positively charged, leading to their aggregation and enlargement over time. In addition, we used Transmission Electron Microscopy (TEM) and Scanning Electron Microscopy (SEM) techniques for the initial characterization of the silver nanoparticles. TEM was used to directly visualize the size of the resulting AgNPs. As shown in Fig. 1, we determined that the AgNPs prepared by this method have a size distribution of 1 nm in average size, with also a narrow size distribution. Thus, both the size and surface charge characteristics of the resulting nanoparticles are considered ideal for their potential therapeutic applications [17, 28].

**Fig. 2:**
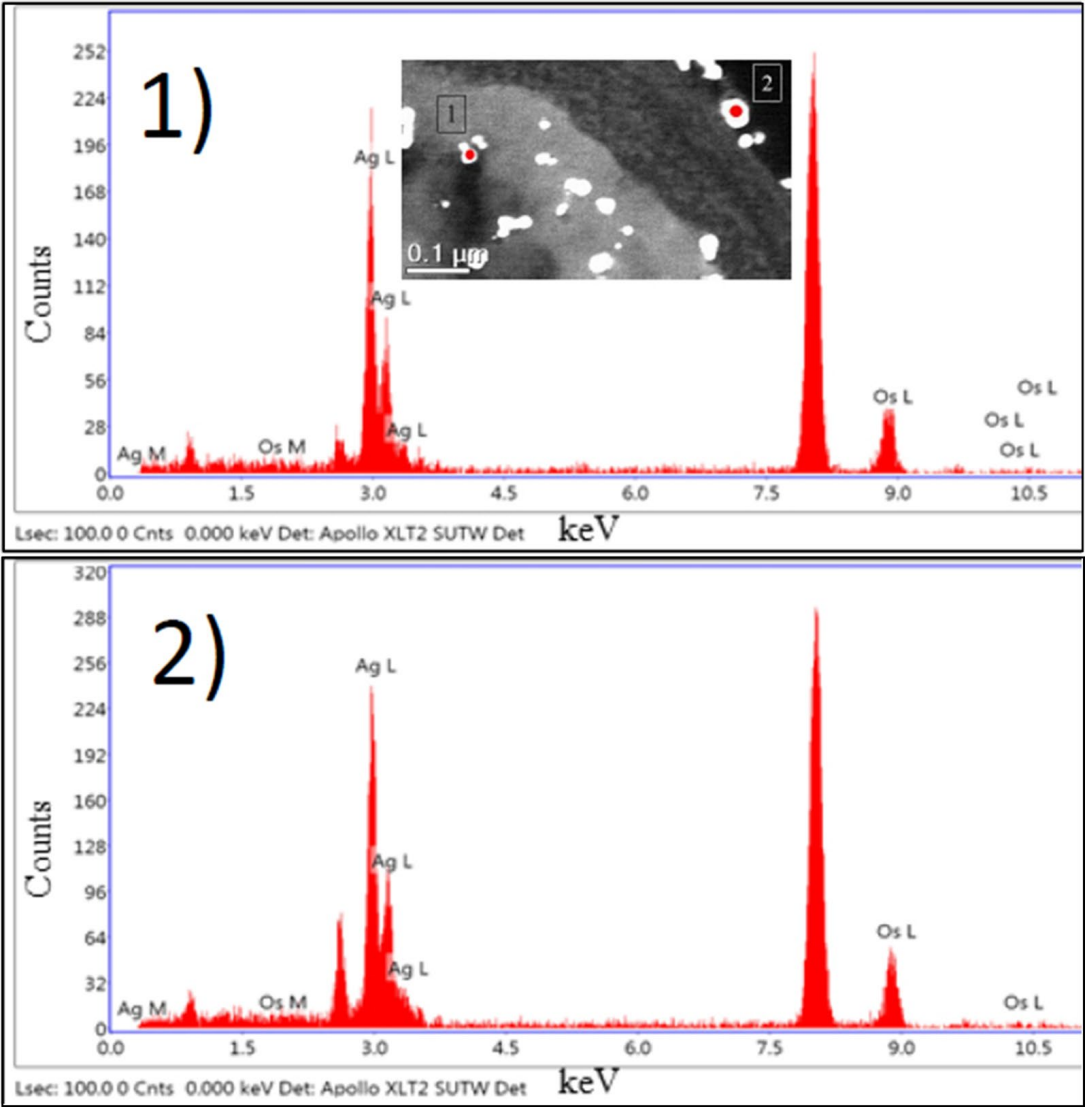
EDS spectra of silver signal *1* inside the yeast cell and *2* on the outer CW. TEM micrograph shows distribution of AgNPs inside the planktonic cell and also on the surface of the outer CW after 24 h incubation. EDS spectra of silver nanoparticles demonstrate the presence of elemental silver signal in the sample

The silver nanoparticles were also characterized on the Candida’s biofilm by SEM and Energy-Dispersive X-ray Spectroscopy (EDS), at a voltage of 20 kV in a Hitachi S-5500 SEM (Additional file 2) and also with an EDAX Apollo XL SDD detector EDX spectroscopy (Mahwah, NJ, USA) (Fig. 2) demonstrating the presence of silver inside and also on the outer CW of the yeast cell by TEM after 24 h incubation.

### Inhibitory effects of AgNPs against *C. albicans* biofilms

**Fig. 3:**
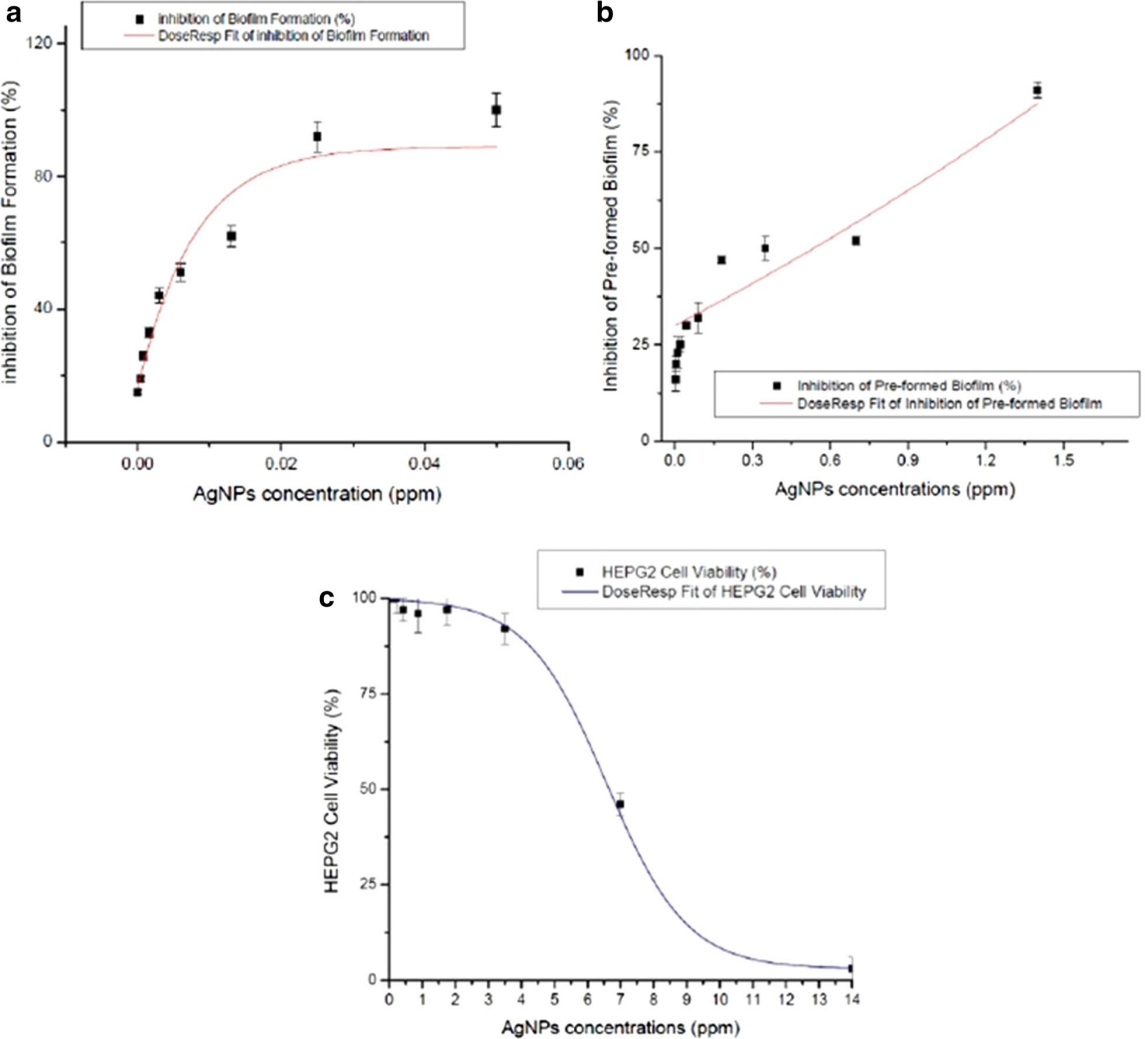
Phenotypic Assays on Biofilm inhibition by AgNPs. **a** Different AgNPs concentrations in two-fold serial dilutions were added to *C. albicans* cells. The IC_50_ values for inhibition of biofilm formation were calculated as AgNPs 0.089 ppm (**b**) AgNPs was also added to preformed (24 h) biofilms. The IC_50_ values for pre-formed biofilm reduction were calculated as AgNPs 0.48 ppm. All experiments were performed in duplicate on three separate occasions. Results represent the means ± standard deviations (SD). **c** The cytotoxic concentration 50 % (CC_50_) of AgNPs was measured in HEPG2 cell line. The experiments were performed in triplicate, and the results are presented as the mean ± SE. The CC_50_ values for cell toxicity were calculated as AgNPs 7.08 ppm

As mentioned before, biofilm formation by *C. albicans* complicates treatment of these infections, as sessile cells within the biofilms display high levels of resistance against most clinically-used antifungals [11, 29]. Thus, there is an urgent need for novel approaches for the prevention and treatment of biofilm-associated candidiasis [11]. In recent years there has been increased interest in the utilization of metal, and in particular silver nanoparticles for the treatment of microbial infections [14, 23, 30], including those associated with a biofilm etiology [31, 32]. Thus, in these studies we used a model of biofilm formation on the wells of microtiter plates in order to examine the inhibitory effects of the AgNPs against *C. albicans*, both for the inhibition of biofilm formation, as well as for their activity against pre-formed biofilms. Results indicated a dose-dependent and potent inhibitory effect of AgNPs on biofilm formation, with a calculated IC_50_ of 0.089 ppm (Fig. 3a). AgNPs also demonstrated efficacy when tested against pre-formed *C. albicans* biofilms; although, as expected, activity was detected at higher concentrations than those required to inhibit biofilm formation, resulting in a calculated IC_50_ of 0.48 ppm (Fig. 3b). Importantly, the concentrations of AgNPs displaying effects against *C. albicans* biofilms are much lower than those at which they demonstrate toxicity, as measured by a standard cytotoxicity assay using human hepatocellular carcinoma (HepG2) cells, resulting in a CC_50_ of 7.03 ppm (Fig. 3c), thereby indicating therapeutic potential.

### Ultrastructural analysis of planktonic *C. albicans* cells after treatment with AgNPs utilizing SEM

**Fig. 4:**
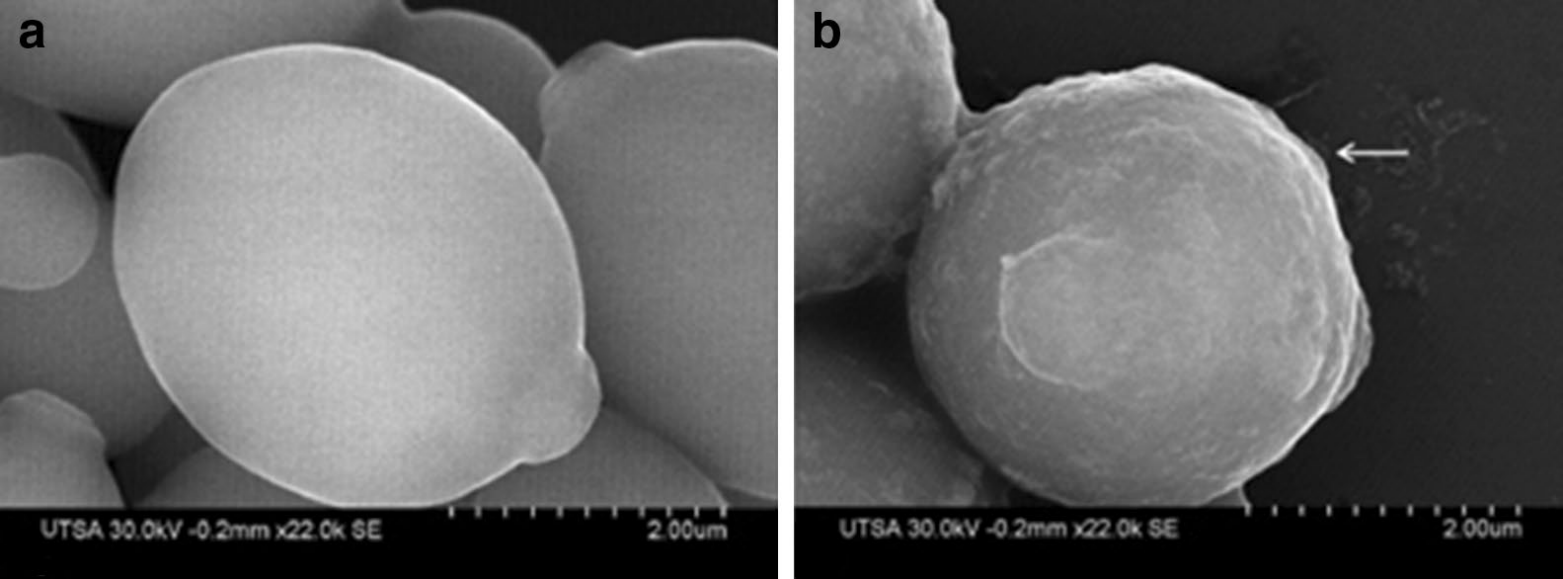
SEM images of planktonic *C. albicans* cells treated with AgNPs. **a** Yeasts without treatment show a smooth surface on the CW and **b** yeast after treatment, the *white arrow* point at rough outer CW. After 24 h incubation and treatment with AgNPs 0.089 ppm

The SEM images a sample by scanning across the specimen’s surface with a high-energy beam of electrons, it is capable of imaging at a considerably enhanced resolution than light microscopes and possesses an improved depth of field on the fungal surface and overall biofilm topology [33]. With this SEM technique the morphologies of cells within the biofilms were studied, both in the absence and in the presence of AgNPs treatment. The ultrastructural differences readily visualized under SEM with AgNPs 0.0089 ppm treatment were major changes in the surface appearance from smooth (Fig. 4a) to rough (Fig. 4b), thus indicating outer CW damage.

### Visualization of fungal biofilms by SEM

**Fig. 5:**
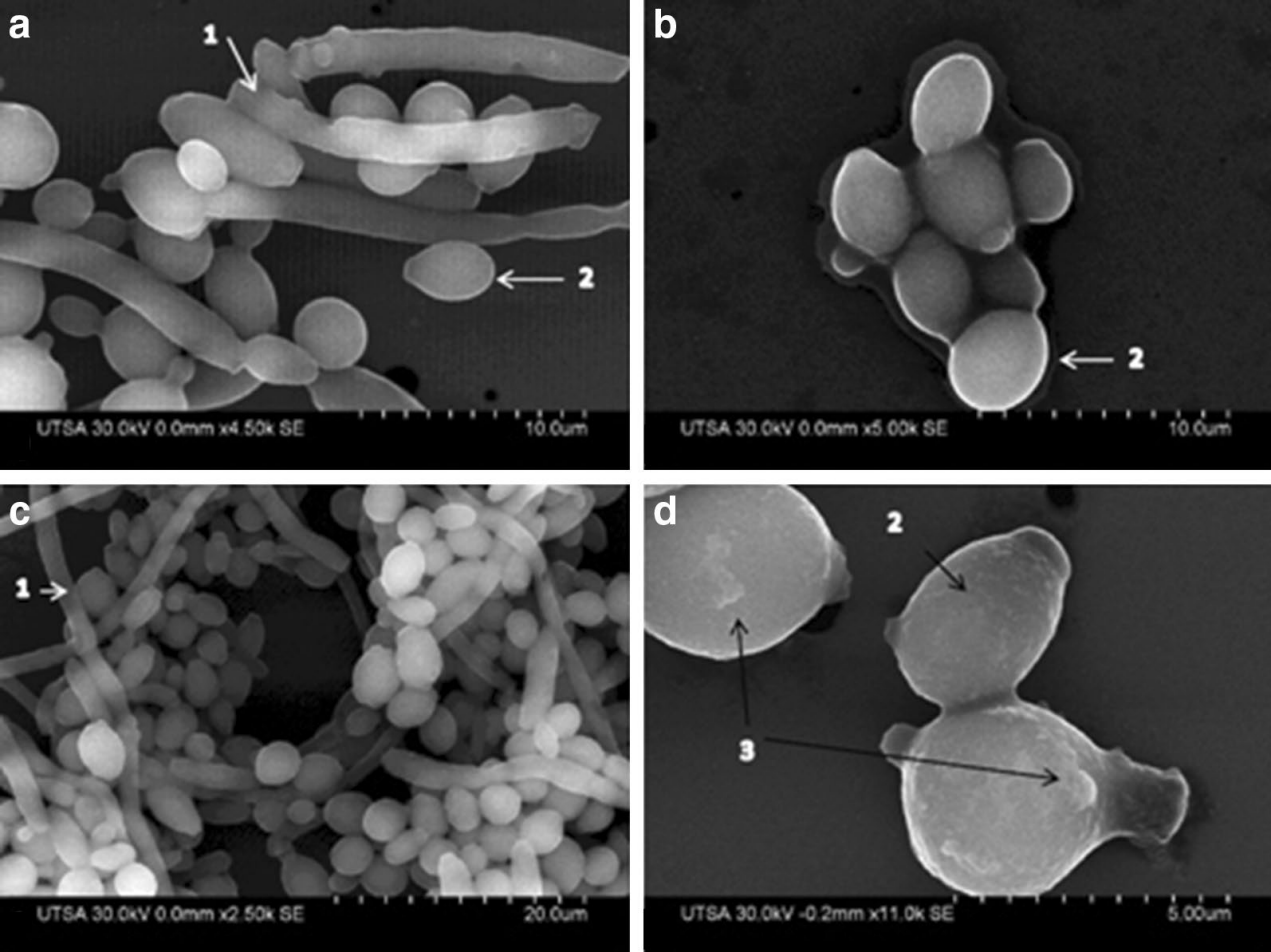
SEM images of biofilm formation of *Candida albicans* inhibited with AgNPs (**a**) and (**c**) biofilm formation without any treatment displaying (*1*) True hyphae and (*2*) Yeast, (**b**) and (**d**) show biofilm formation inhibited by AgNPs after 24 h treatment at 0.089 ppm with inhibition of growth, scarce hyphae showing a disrupted and rough outer CW (as indicated by *black arrows*) in (*3*) disruption on the outer CW

Under biofilm-growing conditions, cultures were treated with AgNPs at a concentration of 0.0089 ppm (Fig. 3) during 24 h. As a result we observed scarce biofilms, which were composed mostly of yeast cells and few filamentous forms. True hyphae were mostly absent from these biofilms, as shown in Fig. 5b, d, treatment with AgNPs was able to inhibit filamentation and subsequent biofilm formation. This is important as both filamentation and development of biofilms represent two of the main virulence factors of *Candida* species [11].

### Visualization of fungal pre-formed biofilms by SEM

**Fig. 6:**
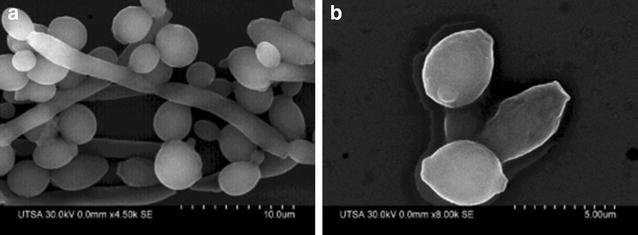
Preformed biofilm of *C. albicans* treated with AgNPs. **a** Preformed biofilm and **b** preformed biofilm after 24 h treatment of AgNPs. Cells were incubated for 48 h and treatment with AgNPs was 0.48 ppm. Treated pre-formed biofilm had almost no true hyphae, and was clearly reduced in number of cells, disruption of the cell-wall is observed in treated pre-formed biofilm

SEM was also used to examine the effect of AgNPs at a concentration of 0.48 ppm, previously determined to be effective by the XTT-reduction assay against pre-formed biofilms (after 24 h incubation). The corresponding images show that the resulting biofilms show scarce cells, which were composed mostly of yeast cells with few pseudohyphae or true hyphae as observed in Fig. 6b as opposed to the pre-formed biofilm in the absence of nanoparticles treatment, which showed a characteristic dense network of hyphae and yeast cells (Fig. 6a).

**Fig. 7:**
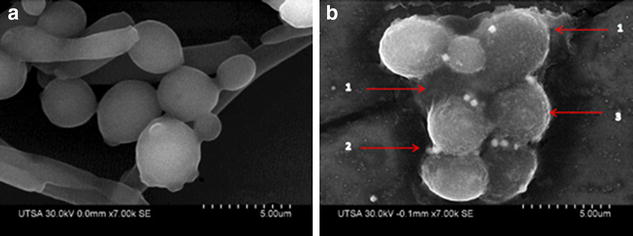
SEM images of C. *albicans* Biofilm. **a** SEM micrograph of a pre-formed biofilm without treatment and **b** preformed biofilm after AgNPs treatment (0.48 ppm) showing; *1* the EPS protecting the yeasts, *2* AgNPs on the EPS aggregating, *3* damage to the CW with no hyphal formation. AgNPs 0.48 ppm are aggregated 24 h after incubation of the biofilm

Importantly, these mature biofilms are encased within a self-produced protective extracellular matrix (ECM) which also imparts most of its resistance properties [34, 35]. Interestingly we were able to document the presence of AgNPs on the surface of the EPS. As in the case of conventional antifungals, these ECM may also contribute to the reduced susceptibility of preformed biofilms against AgNPs as compared to their efficient ability to inhibit the formation of biofilms (Fig. 7b-2), the EDS for silver signal on the biofilm was performed (Additional file 2).

### Ultrastructural analysis of the interaction between AgNPs and planktonic *C. albicans* cells using TEM

**Fig. 8:**
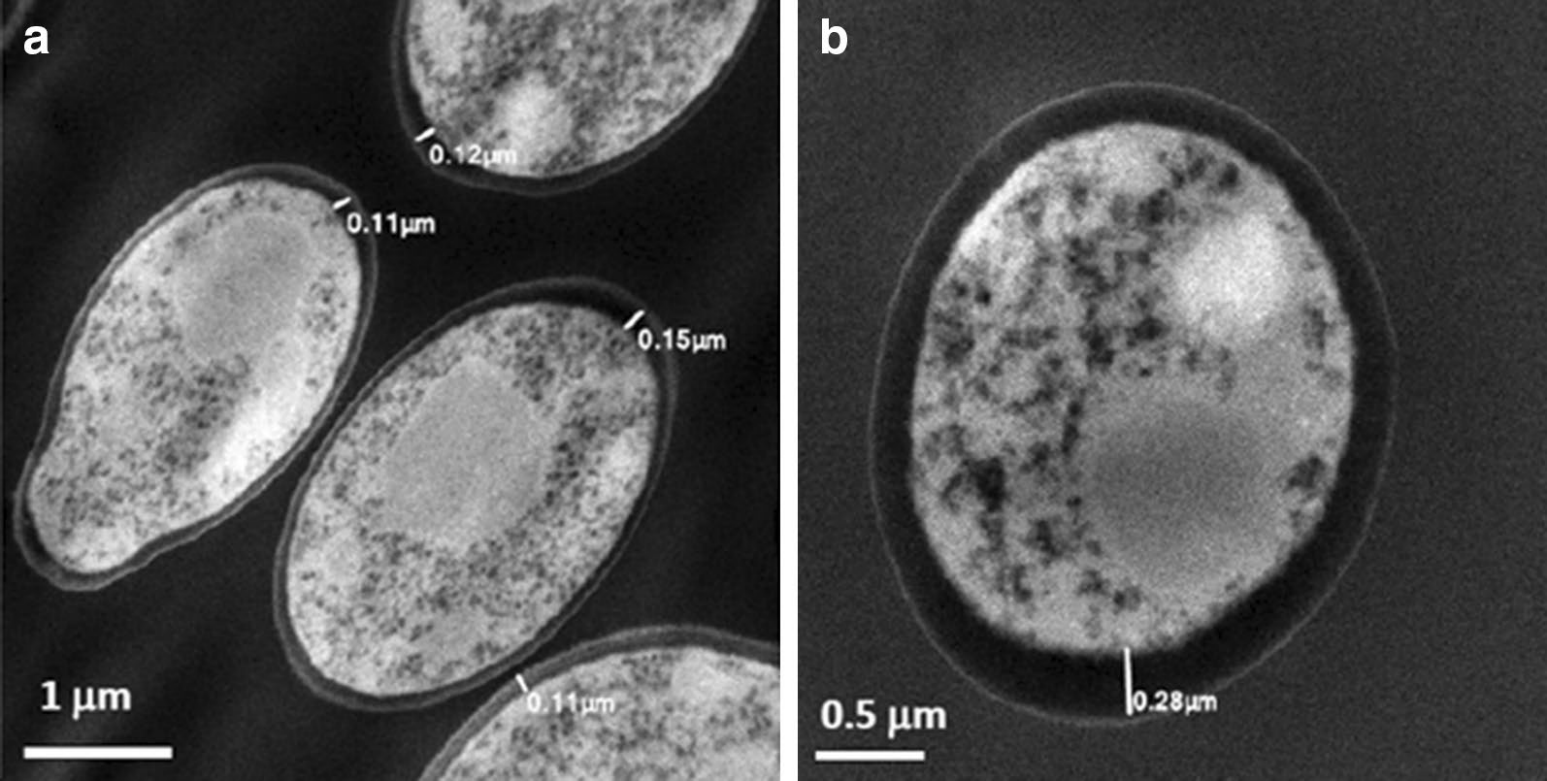
TEM images of planktonic *C.* *albicans* treated with AgNPs. **a** Yeast without treatment, **b** yeast with 24 h treatment with AgNPs (0.0089 ppm), Cells without treatment revealed well-conserved morphological features, with a distinctive cell-wall and cytoplasmic membrane. After treatment with AgNPs enlargement of CW width was evident

Recently, AgNPs have also proven its efficacy against *C.albicans* [21–24] by disturbing the membrane potential and creating pores therefore provoking ion leakage and other materials [20], generating apoptosis [19], and showing ultrastructural changes [18]. Therefore we utilized an atomic resolution analytical microscope (JEM-ARM200F) that achieves a scanning transmission image (STEM-HAADF) resolution of 80 pm, one of the highest for a Cs corrected TEM [36]. This TEM technique was employed to visualize the effects of AgNPs on the ultrastructure of *C. albicans* (Fig. 8). As seen in Fig. 8a, fungal cells grown in the absence of AgNPs displayed well-conserved morphological features, with a typical and distinctive cytoplasmic membrane and cell-wall. In stark contrast, *C. albicans* cells exposed to AgNPs for 24 h (0.089 ppm) were generally enlarged, with alterations in the cell membrane and cell wall, which had increased in thickness and lost its characteristic electron-dense appearance.

**Fig. 9:**
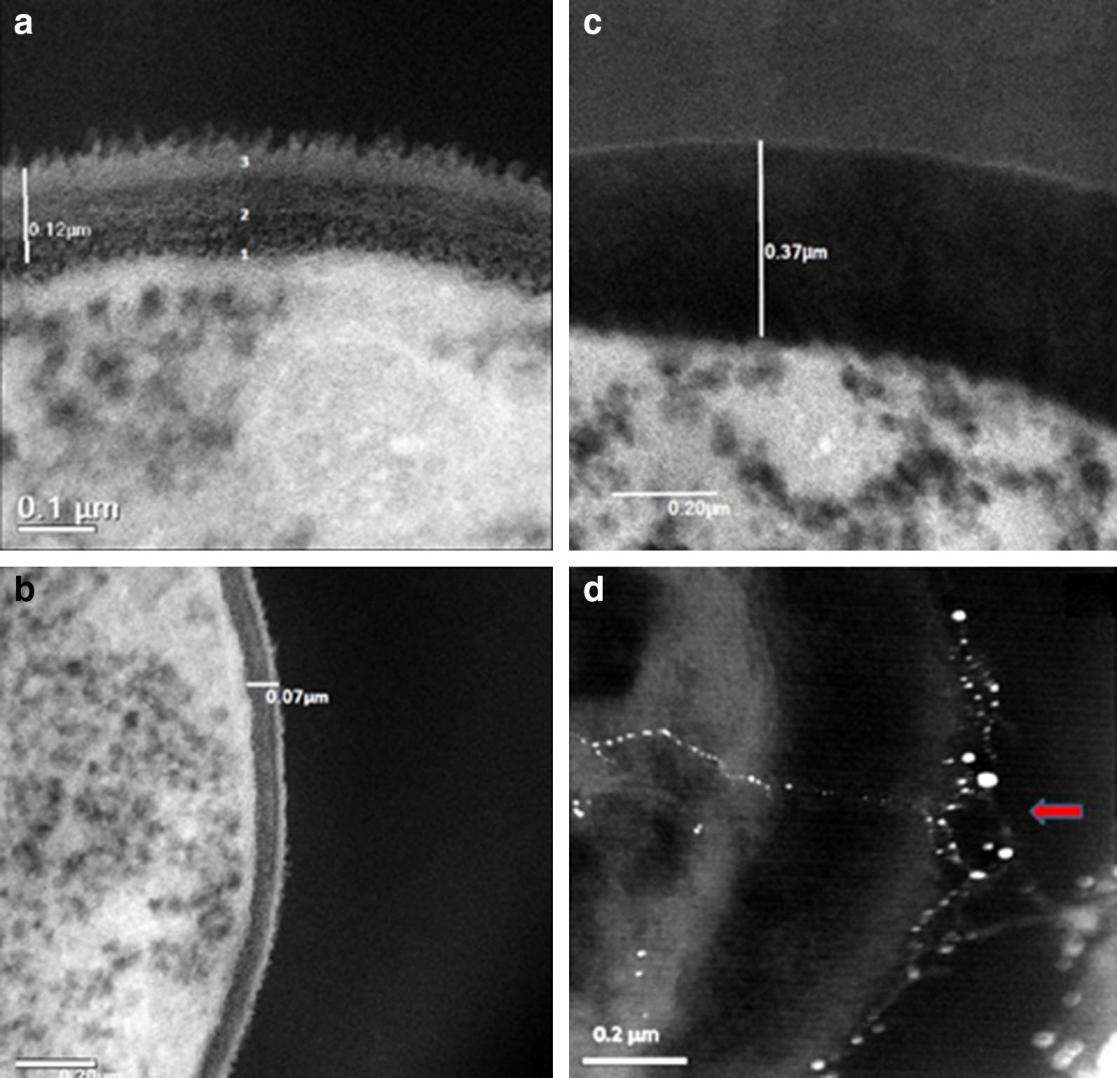
TEM images of the CW of *C. albicans*. **a**, **b** images show yeast without treatment, *3* outer mannoproteins fibrillar layer, *2* inner cell wall layers with preponderance of Glucans and Chitin, *1* plasma membrane. Treated yeast with AgNPs are shown in (**b**) and (**d**) with swollen CW and loose of CW characteristic layers. Also in **d** the *red arrow* indicates an entrance (*pore or hole*), with nanoparticles crossing the cell wall and reaching the plasma membrane after 24 h incubation and treatment with AgNPs 0.48 ppm

The cell wall is essential to nearly every aspect of the biology and pathogenicity of fungi, with pivotal functions in adhesion properties and morphogenetic conversions [37]. In *C. albicans* the cell wall is composed mostly of glucans, chitin and mannoproteins. The cell wall is typically considered a layered structure. Glucans and chitin, the main structural components that supply rigidity to the overall wall structure, appears to be more concentrated in the inner cell wall layer adjacent to the plasma membrane, whereas mannoproteins are present through the entire wall and are particularly abundant at the most external cell wall layers [37], including at the surface which normally exhibits a fibrillar appearance [37]. As seen in Fig. 9a, b, untreated fungal cells show the distinctive cell wall layers and cell membrane, but in treated cells with AgNPs the layers within the enlarged cell wall are difficult to distinguish.

**Fig. 10:**
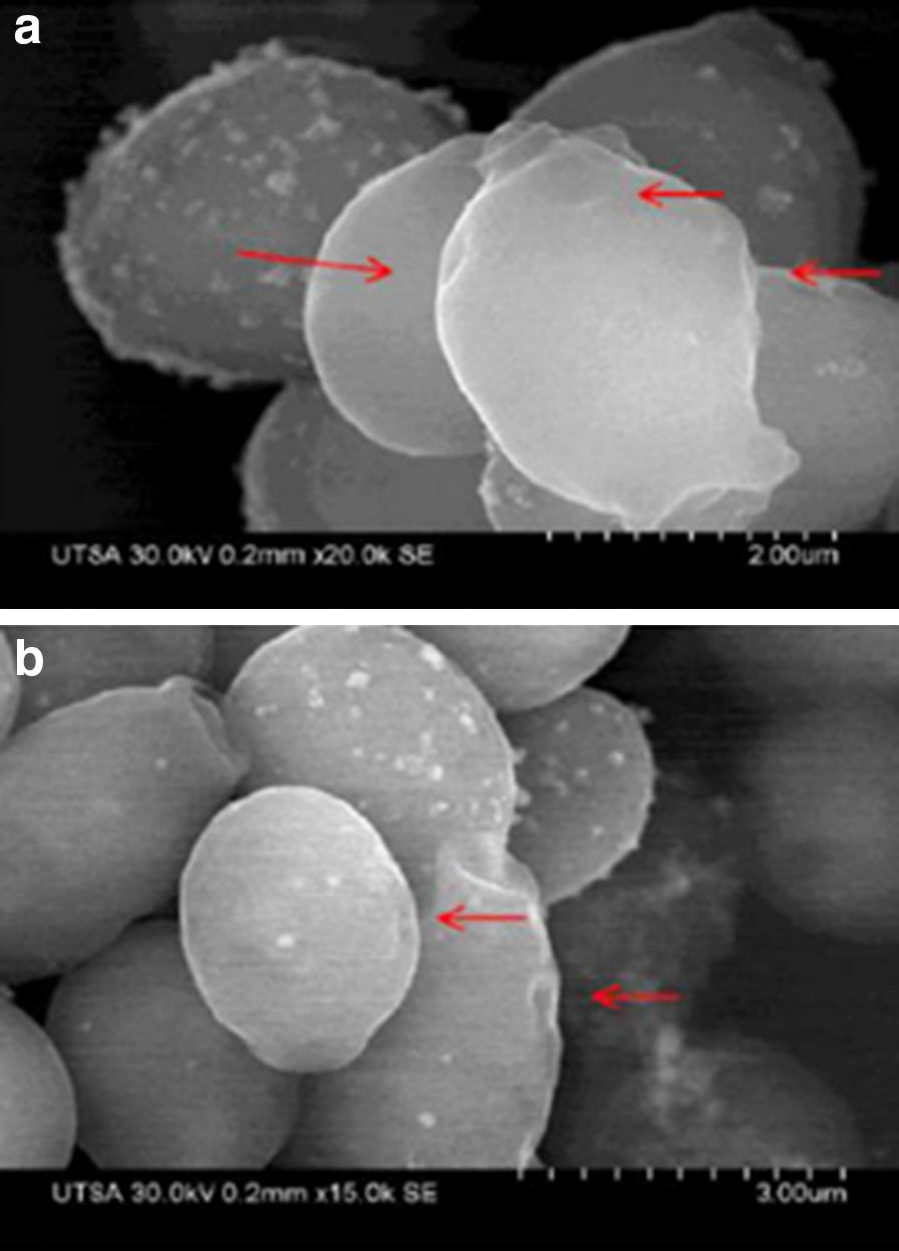
SEM images of treated planktonic yeast. **a**, **b** *C.* *albicans* treated with AgNPs, *red arrows* point at disruption on the outer CW after 24 h incubation and treatment with AgNPs 0.089 ppm

As shown in Fig. 9d (TEM images) and Fig. 10 (SEM images) we found that the interaction of nanoparticles lead to disruption of the outer CW and subsequently produce permeabilization that let smaller AgNPs to get inside the yeast. These observations are consistent with those reported by another research group, which has previously described these holes and pits [20]. Overall, the AgNPs prepared here demonstrated potent activity against biofilms formed by *C. albicans*, one of the most common opportunistic human fungal pathogens. The anti-biofilm effects are via CW disruption; more precisely, they affected survival of both the yeast and the filamentous forms of the fungus when forming a biofilm at an IC_50_ of 0.0089 ppm and also on a preformed biofilm at an IC_50_ of 0.48 ppm (Fig. 3a, b).

AgNPs exert activity against bacteria or fungi via the permeabilization of target cell membranes [38]. The active AgNPs caused severe morphological changes in the fungal cells manifested by disruption of the cell membrane structure (Figs. 4b, 5d, 6b, 9d). This membrane damage was also supported by SEM images where we observed rough membranes (Fig. 4b) and on TEM images we observed changes in diameter and the structure on the outer and inner membrane (Figs. 8b, 9c, d).

Although the chemical arrangement of the mammalian, bacterial and fungal external membranes diverge to a great degree, their membrane main structures are formed by the phospholipids that contain a negatively charged phosphate group [39] incorporated in the hydrophilic head [40], which could be targeted by membrane-disturbing cationic AgNPs [17].

The cytotoxicity of AgNPs on HePG2 cell lines was investigated (Fig. 3c). AgNPs were not cytotoxic to these human cells at concentrations that were effective against *C. albicans* biofilm formation and pre-formed biofilms, thus indicating that their membrane-damaging activity might not target mammalian phospholipids and membranes at fungicidal concentrations.

The active positively-charged AgNPs not only could inhibit the growth of the planktonic form of the fungi, but also were able to prevent the growth and the subsequent activity of the hyphal form in an in vitro coculture model. This indicates that with proper formulation that helps to maintain the activity of the AgNPs might be effective drugs for the treatment or prevention of candidiasis.

The measurement of CW width was tested with TEM images of 100 yeast cells. The mean and standard deviation of the control CW width were estimated to be 105  ±  13 nm, those measurements for yeasts treated with AgNPs 0.0089 ppm were 206  ±  11 nm, p values were statistically significant at <0.05.

## Conclusions

By utilizing microwave-assisted techniques [25] to produce AgNPs we eliminated the requirement of additional reductors, consequently excluding further contamination sources. With this methodology, we obtained clean AgNPs without contaminants for biological tests [26]. With advanced electron microscopy methods we were able to observe that these positively-charged AgNPs disrupt the cell membrane, permeabilizing the outer CW as it becomes rough and distended, changing the CW width due permeabilization that let smaller AgNPs enter the cell. AgNPs were very effective inhibiting biofilm formation by disrupting the CW and inhibiting filamentation of the yeast. Also AgNPs were effective against a pre-formed biofilm in a dose response manner at a non-cytotoxic range. The observations on the inhibition of biofilm are supported by a phenotypic assay [34].

## Methods

### Chemicals and materials

Silver nitrate, AgNO_3_ (99.99 % purity), was obtained from Sigma-Aldrich Corporation (St. Louis, MO, USA) and utilized without further purification. Distilled water was purified using Whatman^®^ 0.2 µm filters.

### Preparation of AgNPs

**Scheme 1:**
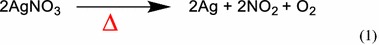
Thermal decomposition of silver nitrate

AgNPs were synthesized through a microwave (MW) induced reaction using an Ethos EZ Digestion System Microwave (Milestone, 2.5 GHz, sensor ATC400) equipment [25, 26]. Briefly, 1.7 g of AgNO_3_ was supplemented to 20 ml distilled H_2_O and introduced in the MW. The solution was continuously irradiated for 15 s at 1000 W. As a result we obtained a clear light yellow solution, demonstrating the synthesis of AgNPs. We tested the solution by ICP spectroscopy obtaining a concentration of 23,000 ppm of silver. The AgNPs were kept in the dark at room temperature to prevent aggregation. Ten microliters of the AgNPs were located on Carbon-coated Cu grids (300-mesh) for further characterization. It is well known that silver nitrate decompose into metallic silver, NO_2_ gas and O_2_ by the action of heat from the MW-irradiation, as represented in the Scheme 1 [41].

Thermal decomposition of silver cations is reduced to metallic silver nanoparticles, yielding NO_2_ gas as a major product. Qualitatively, thermal decomposition is minor below the melting point, but becomes significant around 250 °C and totally decomposes at 440 °C.

### Characterization of AgNPs

#### Electron microscopy characterization

AgNPs were characterized utilizing a high resolution TEM analyses 2010-F JEOL (JEOL Inc, Tokyo, Japan) with a field emission gun operating at 200 kV. HAADF-STEM with resolution of 80 pm, one of the highest for a Cs corrected TEM [36]. Fungal cell images were achieved utilizing microscope in both bright-field (BF) and dark-field (DF) modes. The microscope was operating at 200 kV with a convergence angle of 26 mrad and collection semi angles between 50 and 180 mrad to obtain atomic scale imaging. The probe size used was approximately 0.0089 nm, and the probe current was 22 pA. Energy Dispersive-X-ray Spectroscopy (EDS) was performed using a solid state EDAX EDS detector to identify the AgNPs in the treated biofilm samples. A Hitachi 5500 SEM was used at 30 kV to collect SE images. The Zeta potential (ξ) measurement of AgNPs was determined by dynamic light scattering (DLS) using a Zetasizer NanoZS (Malvern Instruments, Worcestershire, UK) as previously described [42].

#### Strain, media and culture conditions

*Candida albicans* strain SC5314 (a clinical isolate originally obtained from a patient with disseminated candidiasis) [43] was used in this study. For routine culture, cells from stocks stored at −80 °C were propagated by streaking onto Yeast-peptone-dextrose (YPD) agar plates (1 % [wt/vol] yeast extract, 2 % [wt/vol] peptone, 2 % [wt/vol] dextrose, 1.5 % agar), and incubation overnight at 30 °C. From these, a loopful of the overnight *Candida* growth was inoculated into flasks containing 25 ml of YPD liquid media in an orbital shaker at 180 rpm and 30 °C for 14–16 h. Under these conditions, *C. albicans* grows as budding yeast. Biofilms were studied using the 96-well microtiter plate-based method previously reported by our group [34]. Briefly, cells harvested from overnight YPD cultures were washed and diluted in RPMI-1640 supplemented with l-glutamine (Corning-Cellgro) and buffered with 165 mM 3-(*N*-Morpholino) propanesulfonic acid (MOPS, Sigma-Aldrich), with biofilms formed on flat-bottom 96 well microplate (Corning Incorporated) following incubation at 37 °C for 24 h. After biofilm formation, the biofilm firmly attached to the wells were washed twice with phosphate-buffered saline (PBS) to eliminate non-adherent yeasts, and the quantity of biofilm formation determined using semi-quantitative colorimetric technique based on the reduction of 2,3-bis(2-methoxy-4-nitro-5-sulfo-phenyl)-2*H*-tetra-zolium-5-carboxanilide salt (XTT, Sigma) as previously described by our group [34], with ODs determined spectrophotometrically utilizing a microtiter plate reader (Benchmark Microplate Reader; Bio-Rad Laboratories, Hercules, CA). The OD of control biofilms formed in the absence of AgNPs was arbitrarily set at 100 % and data was calculated as percent biofilm inhibition relative to the average of the control wells.

#### Inhibitory effects of AgNPs against ***C. albicans*** biofilms

AgNPs were evaluated for the inhibition of *C. albicans* biofilm formation, as well as for their fungicidal activity against pre-formed biofilms, at different concentrations, ranging from 0.046 to 0.00036 ppm (for biofilm inhibition) and 1.38 to 0.003 ppm (for activity against pre-formed biofilms) in twofold dilutions as previously described [34]. Briefly, to evaluate the effect of the AgNPs in preventing biofilm formation, 50 μL of AgNPs diluted in RPMI media to appropriate concentrations were added to wells containing 50 μL of 2 × 10^6^/mL *C. albicans* cells in a 96 microtiter well-plate. To evaluate the efficacy of AgNPs against pre-formed biofilms, wells were inoculated with 100 μL of 1 × 10^6^/mL *C. albicans* cells, the plates were incubated for 24 h and gently washed with PBS before 100 μL of different concentrations of AgNPs were added. After the addition of AgNPs, the 96 well-plates were covered with parafilm and incubated at 37 °C for 24 h. The plates were then washed twice with sterile PBS to remove non-adherent planktonic cells and processed using the tetrazolium salt (XTT) reduction assay to test the efficacy of the AgNPs preparations. All experiments were performed in duplicate and were repeated at least three times. The IC_50_ (defined as the concentration of AgNPs leading to 50 % inhibition) was calculated from the dose–response curves determined by fitting the data using Origin 8 (OriginLab Corp., Northampton, MA).

#### Cytotoxicity assay

Human hepatocellular carcinoma (HepG2) cells (ATCC#HB-8065) were used to determine the cytotoxicity of AgNPs as in [44]. Briefly HepG2 cells were maintained in minimum essential medium (MEM, Gibco) enriched with 10 % fetal bovine serum (FBS), 1 mM sodium pyruvate (Gibco, Carlsbad, CA, USA), 1× MEM amino acid solution (Sigma-Aldrich), 100 IU mL Penicillin, and 100 mg streptomycin (Cellgro Inc., Herndon, VA). At confluence monolayer-adhered cells were detached and dispersed using 1× Trypsin/EDTA solution (Gibco, Carlsbad, CA, USA). 5 × 10^5^ cells/mL were added 100 µL to each well of the 96-well microtiter plates containing 100 µL of serial two-fold dilutions of the AgNPs. The 96 well plates were incubated for 24 h at 37 °C with CO_2_, and the cytotoxicity was determined using the PrestoBlue cell viability assay [45]. From these data, the CC_50_ value, defined as the concentration of AgNPs leading to 50 % inhibition. All experiments were performed in duplicate and were repeated for three times. The CC_50_ was assessed from the dose–response curves determined by fitting the data using Origin 8 (OriginLab Corp., Northampton, MA).

#### Ultrastructural analysis of the interaction between AgNPs and planktonic ***C. albicans*** cells using TEM

A suspension of *C. albicans* cells (1.5 × 10^8^ cells/ml) prepared from yeast cultures grown for 24 h at 37 °C in YPD were mixed with AgNPs. The samples were then centrifuged for 10 min at 3500 rpm. The resultant pellets were each resuspended in 5 ml of PBS and spun down again for 10 min for washing. After washing two times, fixation of the fungal cells was performed by resuspending each pellet in 1 ml of 4 % formaldehyde and 1 % glutaraldehyde in PBS. After 2 h incubation at room temperature, the samples were stored at 4 °C until they were stained with 1 % osmium tetroxide (OsO_4_) at room temperature. After washing the *Candida* cells with PBS to eliminate excess OsO_4_, a dehydration series was performed with 25, 50, 75, 95 and 100 % ethanol diluted in dH_2_O. The samples were additionally dehydrated with propylene oxide, embedded in a resin (LX112; Ladd Research Industry) and left to harden for 48 h at 60 °C. The resin capsules were cut using an ultra microtome (Leica Ultracut, UCT) and a 45° diamond knife as previously described [42]. Ultrathin sections of approximately 95 nm were obtained and visualized using STEM mode in a JEM-ARM200F (JEOL USA Inc).

Cell wall width measurement (before and after treatment with AgNPs) was performed by DigitalMicrograph (Gatan, Inc., Pleasanton, CA, USA). In this study, descriptive statistics of CW width was conducted by determining mean and frequency distribution, Chi-square, ANOVA, Kruskal–Wallis and non parametric correlation using Origin 8 (OriginLab Corp., Northampton, MA). P value of less than 0.05 was considered significant.

#### Visualization of fungal planktonic cells and biofilms by SEM

For biofilm visualization by SEM, microtiter 96 well-plates with *C.* *albicans* biofilms treated with AgNPs were washed with PBS and fixed with 4 % formaldehyde and 1 % glutaraldehyde in PBS at room temperature. AgNPs were used at a concentration of 0.0089 ppm for inhibition of biofilm formation and at 0.48 ppm for activity against pre-formed biofilms. The samples were rinsed twice (3 min each) in 0.1 M phosphate buffer and then placed in 1 % osmium tetroxide (OsO_4_) for 1 h. The drying process of the samples was performed in a progressive series of ethanol (30 % for 10 min, 50 % for 10 min, 70 % for 10 min, 95 % for 10 min, and absolute alcohol for 20 min). The specimens were then placed on copper grids to be observed with SEM in a Hitachi S-5500.

## Additional files

10.1186/s12951-015-0147-8 Log-normal size distribution histogram shows the average AgNP size as being approximately 1 nm.
10.1186/s12951-015-0147-8 EDS analysis confirmed the presence of silver on the pre-formed biofilm of* Candida albicans*.

## Abbreviations

EPS: Exopolymeric substance
AgNPs: Silver nanoparticles
TEM: Transmission electron microscopy
SEM: Scanning electron microscopy
EDS: Energy-dispersive X-ray spectroscopy
HepG2: Human hepatocellular carcinoma cells
ECM: Extracellular matrix
CW: Cell wall
MW: Microwave
BF: Bright-field
DF: Dark-field
YPD: Yeast-peptone-dextrose
AgNO_3_: Silver nitrate
