# Supplementary figures and images for: Effect of silver nanoparticles on Candida albicans biofilms: an ultrastructural study
Source: J Nanobiotechnology. 2015 Dec 15;13:91. doi: 10.1186/s12951-015-0147-8 (PMC4678641; doi:10.1186/s12951-015-0147-8)

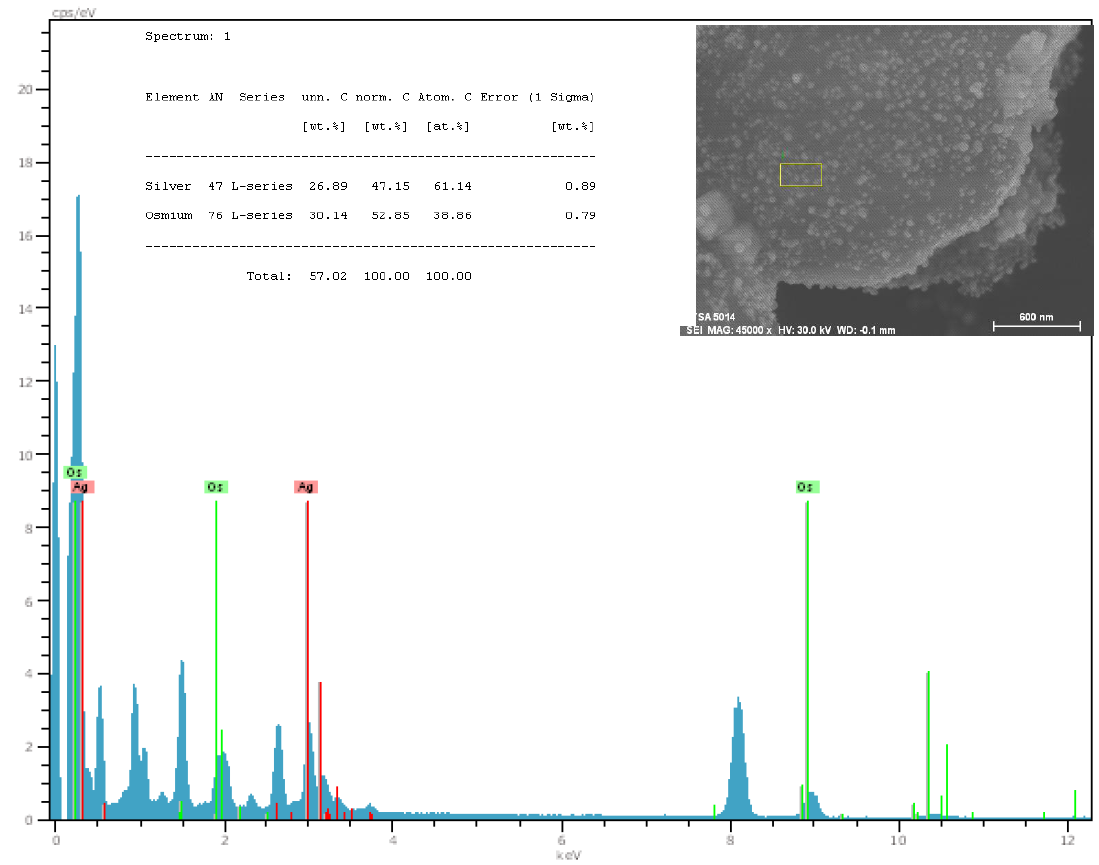

Supplement: Supplementary file 2 — 10.1186/s12951-015-0147-8 EDS analysis confirmed the presence of silver on the pre-formed biofilm of Candida albicans. [file 12951_2015_147_MOESM2_ESM.tif]
